# Intraparotid facial nerve plexiform neurofibroma in a child (case report)

**DOI:** 10.1016/j.amsu.2020.04.022

**Published:** 2020-05-08

**Authors:** Adil Lekhbal, Hicham Lyoubi, Omar Wydadi, Anas Bouzbouz, Reda lah Abada, Sami Rouadi, Mohamed Roubal, Mohamed Mahtar

**Affiliations:** ENT Department, Face and Neck Surgery, Hospital August, 20’1953, University Hospital Centre IBN ROCHD, Casablanca, Morocco

**Keywords:** Neurofibroma, Facial nerve, Parotid gland

## Abstract

**Background:**

Intraparotid facial nerve neurofibromas are benign neoplasms, extremely rare, difficult to diagnose and to manage. Only three pediatric cases have been reported in the literature.

**Case presentation:**

We report the 4th case of a 7-year-old child admitted for a parotid mass without facial palsy, for whom the surgical indication was the increase in volume of this mass, as well as the aesthetical impairment, the surgical exploration found the tumor attached to the lower branch of the division of the facial nerve. The excision of the mass was performed with the sacrifice of the inferior branch of the facial nerve, the trunk and the upper branch of the facial nerve was preserved, the pathological study was in favor of a plexiform neurofibroma.

The patient has presented postoperatively a grade 5 facial palsy in the inferior territory of the facial nerve with a slight recovery 1 year after surgery.

**Conclusion:**

Even though plexiform neurofibromas in the parotid gland are extremely rare, and their diagnosis are not often primary evoked in front of any growing mass of this region, the surgeon must keep in mind the existence of these neoplasms as a differential diagnosis of a parotid tumor.

## Highlights

• Neurofibromas are non-encapsulated tumors that grow inside the nerve that surrounds each nerve fascicle and lengthen them, locally invasive, non-metastatic, highly vascularized, and slow-growing.
• Our patient is the 4th isolated case of intraparotid facial nerve plexiform neurofibroma without neurofibromatosis type 1.
• Preservation of the nerve during surgery is almost impossible and its excision can lead to significant morbidity.
• It is essential that the surgeon keep in mind the existence of these tumors as a differential diagnosis of a parotid tumor.

## 1 Introduction

Peripheral nerve neoplasms include several well-defined clinicopathological entities [1], ranging from benign neoplasms, such as neurofibroma, to high-grade malignant neoplasms, which are often resistant to conventional therapies [2].

Benign peripheral nerve tumors are unusual lesions; eight times out of ten, they are schwannomas, and the other tumors are much rarer, with a wide histopathological diversity [3].

The neurofibroma of the facial nerve in most cases affects the intracranial and intratemporal areas; intraparotid neurofibromas are extremely rare and mainly associated with neurofibromatosis type 1 (NF1) [4].

Neurofibroma is a benign tumor of the peripheral nerve sheath surrounding several nerve fascicles, locally invasive, non-metastatic, highly vascularized, and slow-growing [5].

We report a case of plexiform neurofibroma of the intraparotid part of the facial nerve in a 7-year-old child without NF1, in accordance with SCARE criteria [6], to raise the attention of surgeons to this type of tumor often neglected, but with dramatic consequences for both patient and surgeon.

## 2 Presentation of the case

A 7-year-old child was referred for evaluation and management of a right parotid mass that had been progressively growing for 2 years, without pain, trismus or facial palsy, and without any previous trauma. There was no family pattern or history of neurofibromatosis type 1 (NF1).

Clinical examination showed a soft mass of 4 cm long axis in the right pretragial and parotid region, moving in relation to the superficial and deep planes.

**Fig. 1:**
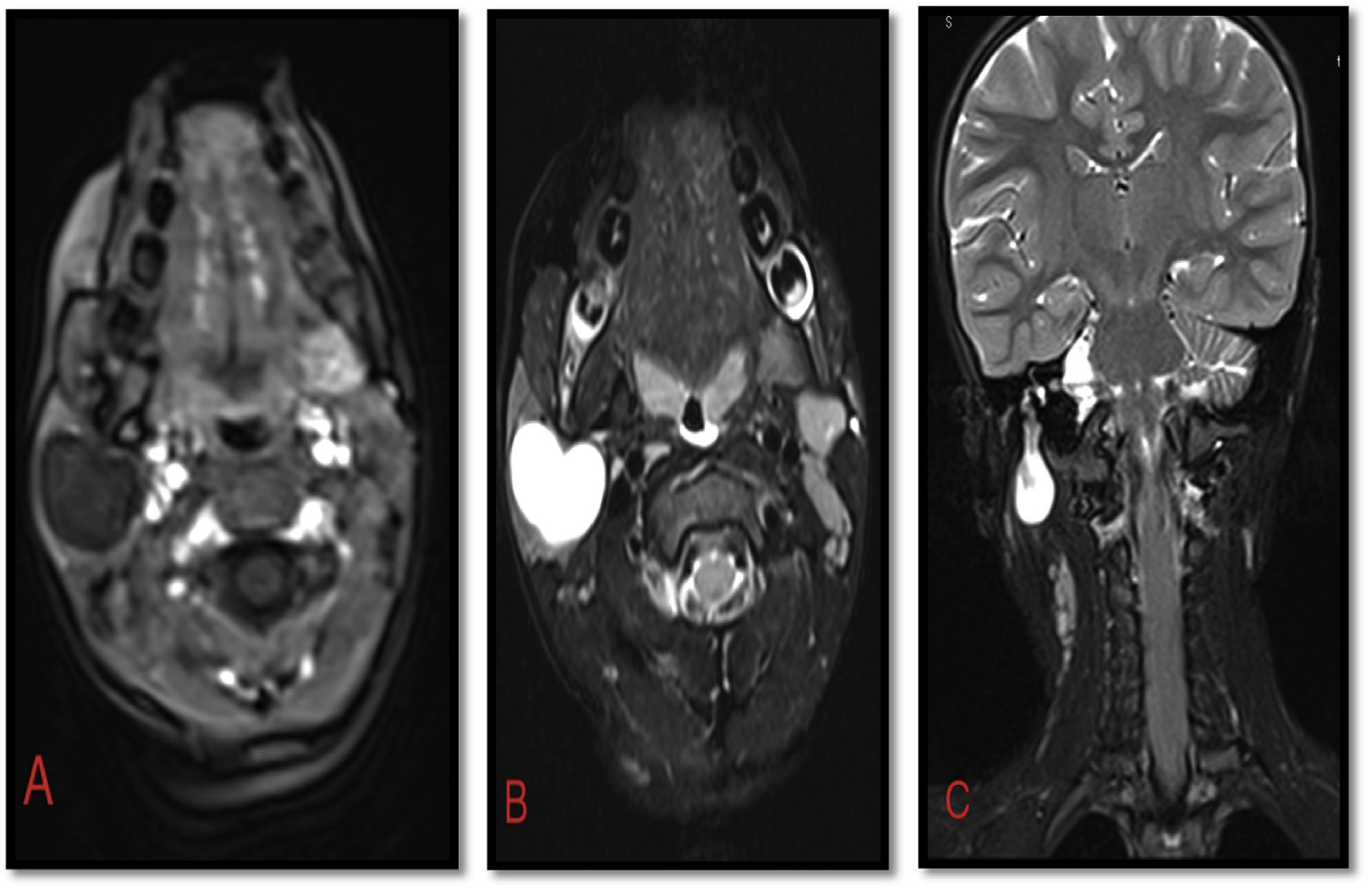
MRI of the parotid gland in sequence T1 (A) axial, T2 (B) axial, and T2 (C) coronal, showing the presence of a polylobed mass with a liquid signal, with an extension towards the skull base most likely related to the facial nerve.

The patient underwent an MRI of the parotid gland (Fig. 1a,b), which revealed the presence of a voluminous well limited polylobed formation located in the two lobes of the right parotid gland, with regular borders, measuring 26 × 25 mm and extending over 46 mm, which was late gradually enhanced after injections to become homogeneous.

Regarding the increase in the volume of the mass and the aesthetic problem, the patient was planned for a parotidectomy. the first step of this surgery consisted of a conventional incision to access to the parotid region, liberation of the posterior part of the gland, and identification of the trunk of the facial nerve. Resection of the superficial lobe was started finding the tumor extension of the inferior branch of division of the facial nerve. The tumor was resected with the sacrifice of the inferior branch of the facial nerve and respect of the continuity of the trunk and the superior branch of the nerve. Nerve reconstruction by anastomosis or transplantation of another nerve was not performed in the same surgical procedure.

**Fig. 2:**
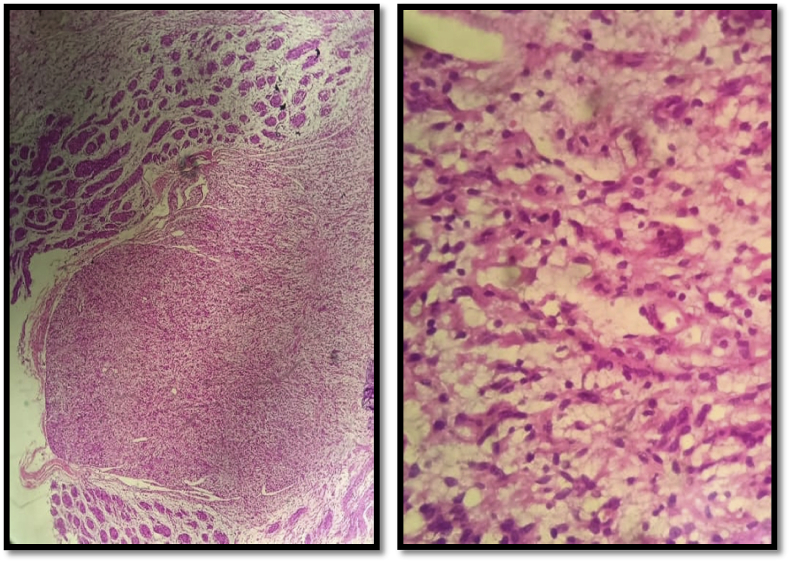
Pathological features of plexiform neurofibroma with Invasion of multiple peripheral nerve fascicles without cellular atypia.

In the pathological study without immunohistochemistry, the tumor was poorly limited, unencapsuled, highly vascularized, containing numerous neoformed nerve threads, and hyperplastic. On the other hand, there was a tumor component, diffuse, moderately cellular, made up of spindle-shaped, thin, endured cells, with poorly defined eosinophilic cytoplasm and round or ovoid nuclei, without atypia or mitosis evoking a plexiform neurofibroma (Fig. 2).

**Fig. 3:**
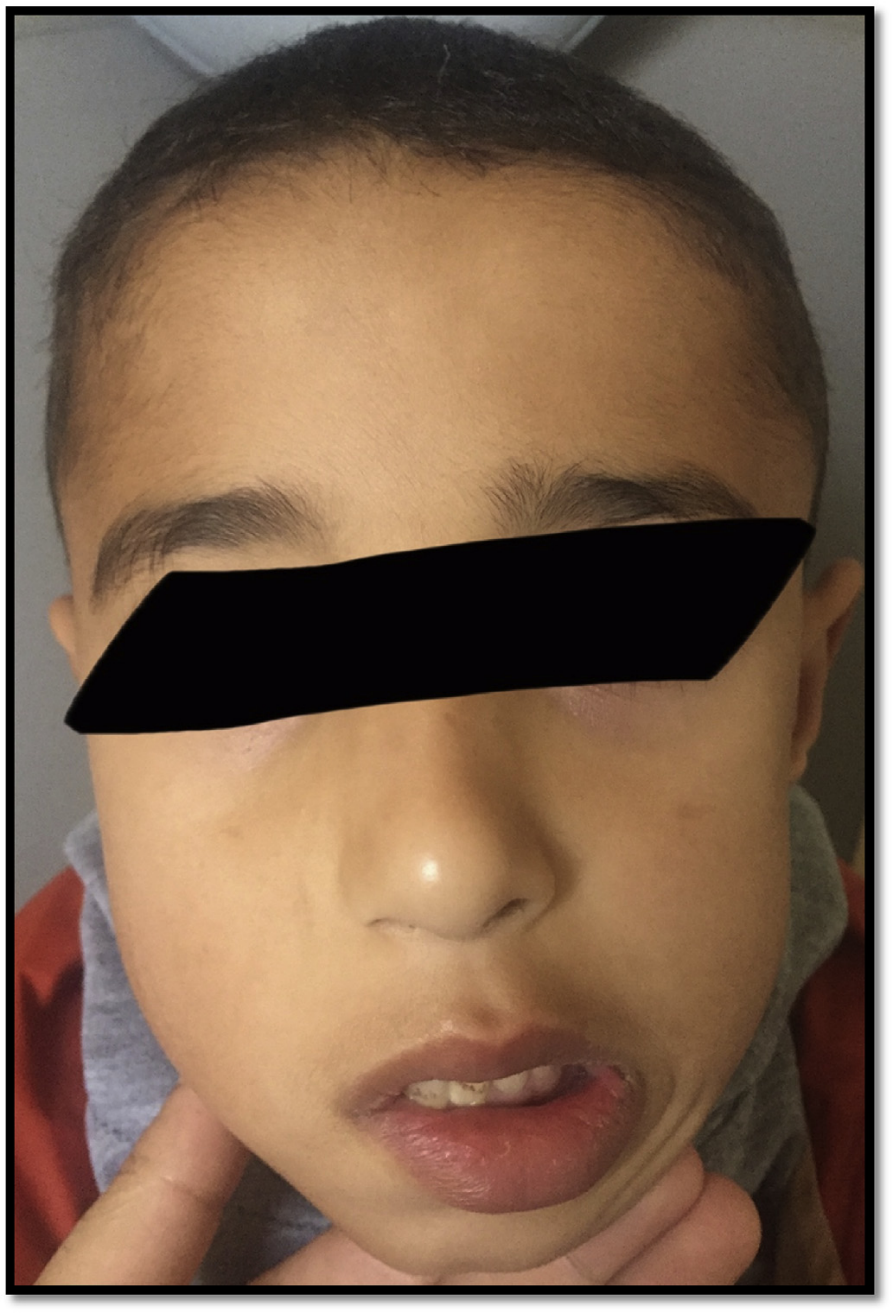
The patient after one year (postoperatively) with an asymmetry in the inferior territory of the right facial nerve.

Postoperatively, the patient presented with grade 5 facial palsy in the inferior territory of the facial nerve, which recovered slightly 1 year after surgery (Fig. 3).

A postoperative review of the MRI scan showed that the mass extended to the skull base (Fig. 1c).

## 3 Discussion

Schwann's cell tumors are classified into two types: schwannomas and neurofibromas, the histopathological differentiation between schwannoma and neurofibromas generally presents little difficulties, schwannomas are well encapsulated tumors, unlike neurofibromas which are non-encapsulated tumors that grow inside the nerve that surrounds each nerve fascicle and lengthen them, it presents as an irregular cylindrical dilatation of the nerve [7]. The plexiform subtype can be recognized by the presence of tortuous and multinodular lesions, and this variant has a high risk of malignant transformation in up to 15% of patients with NF1 [8].

To our knowledge, 12 cases of plexiform neurofibromas of the intraparotid facial nerve have been reported in the English literature, three of which are not associated with NF1 [9,10]. Our patient is the 4th isolated case of plexiform neurofibroma.

Neurofibroma most often affects patients between the ages of 20 and 30, with no sexual preference. In 60–90% of cases, they occur in patients who do not have Neurofibromatosis type 1 (NF-1) [11]. Three types of neurofibromas are described: localized, diffuse and plexiform. Both diffuse and plexiform neurofibromas are often associated with NF-1 [12].

The clinical symptomatology is non-specific, and the patient may present with painless swelling of the parotid region; other symptoms are rare, such as pain, facial spasms, and facial palsy; the masses that cause palsy are large in size, and occur as a result of nerve compression rather than destruction [13].

Peripheral nerve tumors show characteristic images on CT and MRI. On CT scans, neurofibromas shows a very limited mass, which is hypodense due to the presence of Schwann cells [12]. After the injection, neurofibromas generally shows little or no contrast. More than half of the neurofibromas remains hypodense after contrast injection [14]. On MRI neurofibromas show low to intermediate signal intensity on TI, and high signal intensity on T2. After the administration of paramagnetic agent inhomogeneous contrast enhancement is seen in two third of cases although uniform enhancement may be noticed [12].

The differential diagnosis is made with tumors frequently found in this region, and especially with a number of neoplastic and non-neoplastic nerve lesions, the most common of which is schwannoma [5]. In anatomopathology, neurofibroma is poorly limited, not encapsulated, with the absence of thick-walled vessels, Immunohistochemistry is of limited value in this differential diagnosis, as both tumors express S100 protein, but less than schwannoma [5,15,16].

The management of these tumors is controversial when the facial function is preserved before the surgery. Unlike schwannomas which tend to move away the nerves and allow its dissection, in neurofibromas the nerve fibers pass directly into the tumor making them inextirpable and requiring a section of the nerve with reconstruction [17]. On the other hand, May et al. recommends avoiding nerve section when all clinical parameters suggest a benign neurofibroma, meanwhile others, such as Sullivan, have pointed out the possibility of monitoring these lesions with an electroneurography and a CT scan [10]. In our case, an excision of the mass was performed with the sacrifice of the inferior branch of facial nerve division without reconstruction.

For surgical management of the facial nerve, when the tumor is voluminous, a large resection often results in the inability to reconstruct the nerve, due to the small residual distal nerve branches, reconstruction must be performed whenever a great trunk of the nerve is preserved, offering the possibility of a nerve graft, and preferably in the same surgical procedure to avoid the difficulties encountered in identifying the nerve, as the function obtained from a nerve graft is much preferable to no reconstruction [18,19].

## 4 Conclusion

The plexiform neurofibroma of the intraparotid facial nerve is extremely rare. Preservation of the nerve during surgery is almost impossible and its excision can lead to significant morbidity. It is essential that the surgeon keep in mind the possibility of these tumors as a differential diagnosis of a parotid tumor. Given the absence of preoperative clinical signs that may point to facial nerve damage in the majority of cases, an awareness of their existence is necessary for appropriate management of these patients.

Surgery remains the gold standard in the treatment of these locally invasive tumors, however, guidelines need to be established regarding the selection of surgical candidates.

## Consent of patient

The child's mother states that she does not have problems with the publication of her child's cases.

## Funding sources

Funding sources This research did not receive any grant or funding from governmental or private sectors.

## Provenance and peer review

Not commissioned, externally peer reviewed.

## Ethical approval

We've done this work on one case, so we don't need ethics committee approval. Just the patient's consent was taken for the publication of his case.

## Sources of funding

Funding sources This research did not receive any grant or funding from governmental or private sectors.

## Author contribution

Lekhbal Adil: Corresponding author, writing the paper. Lyoubi Hicham: writing the paper. Wydadi Omar: writing the paper. Bouzbouz Anas: writing the paper. Reda Abada: correction of the paper. Sami Rouadi: study concept. Mohamed Roubal: study concept. Mohamed Mahtar: correction of the paper.

## Registration of research studies

Case reports that are not first-in-man do not need register (https://www.research.registry.com/help-and-support/faqs).

## Guarantor

Lekhbal Adil.

## Consent

We took a written consent signed by the child's mother for publication after explaining the rarity of her child's tumor and the scientific interest in its publication.

## Declaration of competing interest

All authors have no conflict of interest or financial support with this article.
