# Supplementary material for: Intraparotid facial nerve plexiform neurofibroma in a child (case report)
Source: Ann Med Surg (Lond). 2020 May 8;55:9–12. doi: 10.1016/j.amsu.2020.04.022 (PMC7229263; doi:10.1016/j.amsu.2020.04.022)
Supplement: Multimedia component 1 [file mmc1.docx]

| **SCARE 2018 Checklist** | | | |
| --- | --- | --- | --- |
| **Topic** | **Item** | **Checklist item description** | **Page Number** |
| **Title** | **1** | INTRAPAROTID FACIAL NERVE PLEXIFORM NEUROFIBROMA IN A CHILD (CASE REPORT) | 1 |
| **Key Words** | **2** | Neurofibroma, facial nerve, parotid gland | 2 |
| **Abstract** | **3a** | Intraparotid facial nerve neuroﬁbroma are extremely rare, difficult to diagnose and manage. Only three pediatric cases have been reported in the literature. | 2 |
|  | **3b** | We report the 4th case of a 7-year-old child admitted for a parotid mass where the surgical indication was established in front of the persistence and the increase of his size as well as the aesthetical impairment |  |
|  | **3c** | at the surgical exploration, the tumor was at the expense of the lower branch of division of the facial nerve. An excision of the mass was performed with sacrifice of the inferior branch of the facial nerve, and respect for the continuity of the trunk and the upper branch of the nerve. The patient has presented postoperatively a grade 5 facial palsy in the inferior territory of the facial nerve with a slight recovery 1 year after the surgery. |  |
|  | **3d** | Conclusion: The surgeon must have the fear of a facial nerve tumor in front of any intraparotid mass, their knowledge is the key to a good management. |  |
| **Introduction** | **4** | Benign peripheral nerve tumors are unusual lesions, eight times out of ten are schwannomas, the other tumors are much rarer, with a wide histological diversity [1].  The neurofibroma of the facial nerve in most cases affects the intracranial and intratemporal part. Intraparotid neurofibromas are extremely rare and mainly associated with neurofibromatosis type 1 (NF1) [2].  Neurofibroma is a benign tumor of the peripheral nerve sheath surrounding several nerve fascicles, locally invasive, non-metastatic, highly vascularized, and slow-growing [3].  We report a case of plexiform and diffuse neurofibroma of the intraparotidial part of the facial nerve in a child without NF1, to raise the attention of surgeons to this type of tumor often neglected, but with dramatic consequences for both the patient and the surgeon. | 2 |
| **Patient Information** | **5a** | 7-year-old child, male, from Morocco | **3** |
|  | **5b** | a right parotid mass that had been progressively growing for 2 years, without pain, trismus or facial palsy |  |
|  | **5c** | No previous trauma, and no family pattern or history of neurofibromatosis type 1 (NF1) |  |
|  | **5d** | Drug history: no drug use in the past  family history including any relevant genetic information: absence of particular family histories, and lack of information about the patient's genetic profile.  psychosocial history: no particular psychosocial history |  |
| **Clinical Findings** | **6** | Clinical examination showed a soft mass of 4 cm long axis in the right pretragial and parotid region, moving in relation to the superficial and deep planes. | 3 |
| **Timeline** | **7** | Delay from presentation to intervention: 2 years | 3 |
| **Diagnostic Assessment** | **8a** | The patient underwent an MRI of the parotid gland (figure 1a,1b), which revealed the presence of a very limited voluminous formation in the two lobes of the right parotid gland, with regular contours, and polylobed, measuring 26*25 mm and extending over 46 mm | 3 |
|  | **8b** | We didn't do the Fine needle aspiration, and MRI slices don't indicate pathology. |  |
|  | **8c** | This tumor is very rare, and other diagnoses are the most common, such as pleomorphic adenoma |  |
|  | **8d** | Prognostic characteristics when applicable (e.g. tumour staging or for certain genetic conditions). Include relevant radiological or histopathological images in this section. |  |
| **Therapeutic Intervention** | **9a** | Pre-intervention considerations: no | 3 |
|  | **9b** | -the type of intervention deployed: parotidectomy with sacrifice of the inferior branch of the facial nerve.  -the reasoning behind this treatment offered: n view of the increase in the size of the mass and the aesthetic damage  concurrent treatments: monitoring |  |
|  | **9c** | Intervention details: after a conventional incision for a parotidectomy and finding the facial nerve for dissection the mass was adherent to the inferior branch of the facial nerve.  a frozen section biopsy confirmed the diagnosis of plexiform neurofibroma. The tumor was resected with sacrifice of the inferior branch of the facial nerve and respect of the continuity of the trunk and the superior branch of the nerve. |  |
|  | **9d** | the operation was performed by a specialist ENT surgeon with 2 years of experience. |  |
|  | **9e** | Changes – if there were any changes in the interventions: no |  |
|  | **9f** | Post-intervention considerations: no particular considerations |  |
| **Follow-up and**  **Outcomes** | **10a** | The histological study revealed a plexiform and diffuse neurofibroma (Figure 2) with a richly vascularized tumor proliferation containing numerous neoformed nerve threads, without atypia or mitosis, and poorly limited in periphery.  Postoperatively, the patient presented with grade 5 facial palsy in the inferior territory of the facial nerve, which recovered slightly 1 year after surgery | 4 |
|  | **10b** | 12-month follow-up. |  |
|  | **10c** | Intervention adherence/compliance: as a result of facial paralysis a rehabilitation of facial function was required, the patient agreed to perform kinesetherapy twice a week. |  |
|  | **10d** | Complications: grade 5 facial palsy in the inferior territory of the facial nerve.  A post-operative review of the MRI scan showed that the mass extended to the skull base (Figure 1c). |  |
| **Discussion** | **11a** | To our knowledge, 12 cases of plexiform neurofibromas of the intraparotid facial nerve have been reported in the English literature, three of which are not associated with NF1 [6, 7]. Our patient is the 4th isolated case of plexiform neurofibroma, and the 2nd case described in children | 4 |
|  | **11b** | Weaknesses and limitations in your approach to this case: the absence of MRI sections which show that the tumor follows the path of the facial nerve in order to take precautions before the operation, and the failure to perform fine needle aspiration which is poorly developed in our country |  |
|  | **11c** | The management of these tumors is controversial when the facial function is preserved before the surgery, unlike schwannomas which tend to displace the nerves and allow its dissection, in neurofibromas the nerve fibers pass directly into the tumor making them inextirpable and requiring a section of the nerve with reconstruction [9]. On the other hand, May recommends avoiding nerve section when all clinical parameters suggest a benign neurofibroma, others, such as Sullivan, have pointed out the possibility of monitoring these lesions with an electroneurography and a CT scan [7] |  |
|  | **11d** | Plexiform neurofibroma of the intra parotid facial nerve is extremely rare. Preservation of the nerve during surgery is improbable and excision may result in significant morbidity |  |
|  | **11e** | It is essential that the ENT surgeon keep these tumors in mind in the differential diagnosis of a parotid tumor. |  |
| **Patient Perspective** | **12** | Before the operation the child's parents signed an agreement about the risks of the surgery, and after the operation the parents accepted the results of the surgery after explanation of the origin of the tumor. | 2 |
| **Additional Information** | **14** | 6. Conflict of Interest:  All authors have no conﬂict of interest or ﬁnancial support with this article.  7. Funding sources:  Funding sources This research did not receive any grant or funding from governmental or private sectors. | 6 |
|  |  |  |  |
